# Platform process for production of monoclonal antibodies for research purposes – improvement option

**DOI:** 10.1186/1753-6561-5-S8-P44

**Published:** 2011-11-22

**Authors:** Joern Meidahl Petersen, Claus Kristensen

**Affiliations:** 1 Biopharm Manufacturing Development, API Support, Gentofte, Denmark, DK-2820; 2 Biopharmaceutical Research, Mammalian Cell Technology, Maaloev, Denmark, DK-2760

## Background

In 2006 Novo Nordisk decided to invest in building a pipeline within inflammatory disease management. To support this strategy the cell culture units had to establish technology for expression and production of monoclonal antibodies in CHO cells. A number of technology providers at that time offered proven platform processes for this purpose. It was decided to in-license one of these technology platforms, the one developed at Lonza Biologics, and focus research resources on product innovation rather than development of an in-house production system. The platform has now been fully implemented and the work flow optimised and standardised.

## Platform process review

The platform process comprises:

• Host cell line/expression system

• Medium/feeds (chemically defined – animal derived component free)

• Process parameters

• Scale-down model (shake flask, 100 ml working volume).

**Figure 1:**
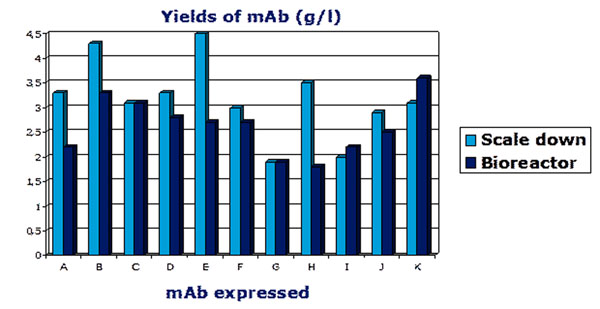
Yields of 11 anitbodies in shake flask and bioreactors.

The platform process has been applied for cell line development and antibody production for R&D purposes for five years. During this period 11 monoclonal antibodies have been transferred from laboratory scale to pilot plant production. The performance of the process platform across projects has been reviewed. The scope of the review was to compare yields obtained in the scale down model and yields obtained in the bioreactor process for all 11 antibodies. Figure 1 shows the results of the review.

In conclusion the review show that

• The cell lines are yielding 1.9 – 4.5 g/l mAb.

• The scale down model is predictive for the bioreactor process.

## Evaluation of an improvement option

An upgrade of the medium, feeds and process protocols was offered by Lonza Biologics. A β-version of the latest process from Lonza has been tested and compared to the previous version in a study including five cell lines. The experiments were carried out in 100 ml shaker flask and the scale down version of the process was applied. A comparison of the two fed batch procedures is shown below:

Current version: Version 6

Medium and feeds CD-ACF

Two feed solution

• Feed rates based on

  ○ Viable Cell Density

  ○ Residual glucose

New version: Version 8

Medium and feeds CD-ACF

Five feed solutions

• Feed rates based on

  ○ Viable Cell Density

  ○ Residual glucose

  ○ Time

The results of the study are compiled in table 1.

**Table 1:** Result of the comparison of the two versions of the fed batch process.

mAb	Current version: Version 6			New version: Version 8		
	
	ICA*^)^	Lactate	mAb	ICA	Lactate	mAb
	10E6 viable cell days/ml	mmol/l	g/l	10E6 viable cell days/ml	mmol/l	g/l
C	108	57	3.5	92	28	7.1
D	103	61	2.2	90	29	5.2
E	162	65	5.5	104	17	9.0
H	100	55	3.2	120	25	7.0
J	148	62	3.2	164	21	4.3

*^)^*ICA: Integrated Cell Area.*

The comparison of the β-test of improved platform process and the current process showed:

• mAb yields improved 1.3 – 2.4 fold

• No change in integrated cell area

Improved lactate control may be the key to the yield improvement.

